# Biofouling Resistance Improvement in Membrane-Based Secondary Effluent Treatment: A Focus on Membrane Surface Modification by Graft Polymerization with 3-Allyl-5, 5-Dimethyl Hydantoin

**DOI:** 10.3390/membranes15100314

**Published:** 2025-10-15

**Authors:** Godwill Kasongo, Aude Minang Nkombe, Mujahid Aziz

**Affiliations:** Environmental Engineering Research Group (EERG), Department of Chemical Engineering, Cape Peninsula University of Technology (CPUT), Chemical Engineering Building, Bellville, Cape Town 8001, South Africa

**Keywords:** biofouling, flux decline, graft polymerization, membrane modification, municipal wastewater, reverse osmosis

## Abstract

The implementation of wastewater management strategies and wastewater treatment techniques, such as reverse osmosis (RO), has been increasing to promote environmental sustainability and reduce freshwater consumption. Municipal secondary effluent is a promising source for reuse and reducing the strain on freshwater consumption. Still, its diverse foulant composition promotes the fouling of polyamide RO membranes, leading to performance decline. In this study, 3-allyl-5,5-dimethylhydantoin (ADMH) was grafted onto thin-film composite RO membranes at varying concentrations via graft polymerization. The membranes were tested against foulant solutions of *E. coli* and *S. aureus*, as well as organic and inorganic foulant solutions mimicking the fouling activity of municipal wastewater secondary effluent. Biofouling tests showed improved mortality ratios—58.9% against *E. coli* and 37.4% against *S. aureus*—along with fouling deposition rates of 3.7–8.9% and flux recovery ratios of 69.2–96.9%. Although surface hydrophilicity increased with ADMH concentration, fouling resistance was optimal at a moderate concentration. Resistance to organic and inorganic foulants did not show similar improvement, highlighting the importance of the foulant type in determining overall membrane performance.

## 1. Introduction

Increasing water scarcity is a global issue resulting from population growth and rising pollution [1]. Therefore, the focus has been on implementing wastewater management solutions and treatment techniques to create global sustainability [2]. Municipal secondary effluent is a consistent and suitable source for advanced treatment and reuse, supporting tertiary processes aimed at producing high-quality water [3]. Tertiary treatment is an additional process designed to reduce further organics, turbidity, bacteria, and other contaminants in wastewater. Membrane technology, specifically reverse osmosis (RO), appears to be one of the most widely used methods for reusing water from various wastewater sources. However, it presents challenges, the most serious being fouling [1,4]. Fouling of the RO membranes causes a decline in permeate flux, lowers permeate quality, and creates a significant economic impact on the overall process due to the cost of maintenance or replacement, as well as the higher pressure required to overcome the flux decline [5,6]. Biofouling is a severe form of membrane fouling whose control and prevention remain the most difficult challenge facing the operations of polyamide membrane systems, despite advances in membrane performance over the last 45 years [7].

A variety of measures have been investigated for fouling control, including pretreatment of feed water and use of disinfectants [8]. However, the biofouling of RO membranes cannot be significantly reduced by pretreating the feed water due to the self-replicating nature of microbial cells [7]. The use of disinfectants, such as chlorine, degrades the membrane and affects its performance in long-term operations [9]. Different methods, such as membrane surface modification techniques, have been studied over the past few years to control fouling. They consist of physically or chemically modifying the membrane surface morphology and characteristics to improve performance [10].

Chemical grafting is a membrane surface modification technique that improves the performance of the membrane and its fouling resistance [4,11]. However, several proposed PA RO membrane modification methods have been reported to enhance specific properties, such as fouling resistance, but they also have drawbacks, including reduced permeability and solute rejection. Ansari et al. [9] found that introducing both polyacrylic acid and graphene oxide to the RO membrane surface improved hydrophilicity; however, the modified membranes exhibited a slightly lower resistance to microbial fouling. Yang et al. [12] modified a polyamide RO membrane using 2-carboxyethyl acrylate (CAA) and cationic [2-(acryloyloxy)ethyl] trimethyl ammonium chloride (TMA). It was observed that the modification enhanced membrane biofouling resistance, while salt rejection and permeability decreased by approximately 20% and 15%, respectively. Recent studies have advanced antifouling membrane design through PEG-based, zwitterionic, and N-halamine modifications. However, it has been reported that PEG layers are prone to oxidative degradation over time [13]. High-concentration zwitterionic coatings achieve excellent hydration and fouling resistance but often require complex synthesis and exhibit limited long-term stability [14]. In a recent study, N-halamine coatings demonstrated strong antibiofouling performance; however, their biocidal capacity declined after repeated recharging cycles [15].

Hence, there is a need to develop PA RO membranes with higher resistance to fouling, particularly biofouling, while maintaining or improving the membrane’s water flux and salt rejection. On the other hand, surface modification must be tailored to allow for selective targeting of specific foulants, as each type of foulant may require a different modification approach to mitigate fouling effectively.

This study evaluates the fouling resistance of a polyamide RO membrane by modifying its surface with 3-allyl-5,5-dimethyl hydantoin (ADMH). The application of the modified membranes is evaluated against model foulants that mimic fouling activities in the secondary effluent of municipal wastewater treatment plants.

Research on ADMH grafting for polyamide RO membranes has demonstrated its potential to enhance chlorine resistance and antibacterial activity [16,17]. However, the field often lacks a comparative perspective, with many studies focusing on a single foulant type, leaving their performance against other prevalent foulants unexplored. The practical challenge, as discussed by Sisay et al. [18], is that surface modifications typically create a complex interplay of properties, in which enhancing one characteristic, such as hydrophilicity, may inadvertently alter another, such as surface charge. This work systematically evaluates ADMH grafting across a range of concentrations (0.2–0.8 mol L^−1^) against a panel of individual foulants encompassing organic, inorganic, Gram-positive, and Gram-negative bacterial foulants, providing clearer insight into how biofouling benefits and fouling resistance correlate with membrane performance.

## 2. Materials and Methods

### 2.1. Materials and Reagents

XLE-4040, a spiral-wound thin-film composite polyamide RO membrane, was obtained from Dow Chemical Company. Sodium bicarbonate, humic acid, potassium hydroxide (KOH), 5.5-dimethyl hydantoin (DMH), allyl bromide, methanol, initiator 2, 2-azobis (isobutyramidine) hydrochloride (AIBA), and glycerol were obtained from Sigma Aldrich (Pty) Ltd., Johannesburg, GP, South Africa. All chemicals were of analytical grade and used without further purification. Nutrient broth powder and nutrients were purchased from Lasec SA (Pty) Ltd., Cape Town, WC, South Africa. Deionized water with a conductivity of less than 5 μS cm^−1^, obtained from a Millipore water purification system in the lab, was used for solution preparation, membrane rinsing, and conservation.

### 2.2. ADMH Synthesis

The synthesis of ADMH was done according to the Gabriel reaction [16]. A solution of 2.8 g (0.05 mol) of KOH in 25 mL of deionized water was prepared, and 6.4 g (0.05 mol) of DMH was added to the solution. Another solution, B, was prepared by mixing 4.4 mL (0.05 mol) of allyl bromide with 10 mL of methanol. Solutions A and B were combined, and the mixture was stirred at 60 °C for 2 h. The solution was cooled and dried under reduced pressure at approximately 25 °C. The resulting solid was recrystallized from petroleum, and multiple batches of ADMH were prepared to produce an aqueous solution for membrane modification.

### 2.3. Membrane Surface Modification

**Figure 1:**
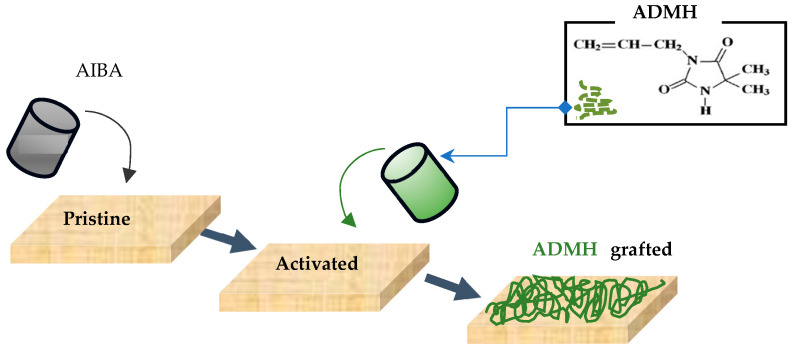
Graphical illustration of the RO membrane surface modification process used in this study.

The modification procedure was like the method employed by Wei et al. [16] adapted to the procedure detailed below. Figure 1 graphically illustrates the modification procedure. A modifying apparatus was constructed with an inner frame and an outer frame, with solution circulation driven by a peristaltic pump. The membrane pieces were taped to the inner frame of the modifying apparatus to ensure that the modifying agent solution was in direct contact with the membrane’s active surface. The aqueous solution of the initiator AIBA was prepared at a mass concentration of 0.02 wt % (i.e., a mass of 0.02 g in 100 mL), while the grafting monomer ADMH solutions were at different concentrations of 0.2 mol L^−1^, 0.4 mol L^−1^, 0.6 mol L^−1^, and 0.8 mol L^−1^, prepared using 2.5 g, 5 g, 7.5 g, and 10 g dissolved in 100 mL of water, respectively. The initiator solution was circulated through the membrane apparatus, and excess solution was removed by gently drying the membrane under a lab-supplied nitrogen stream for 2 min. The apparatus was then flushed with deionized water for 15 min. The ADMH solution was pumped through the system for 20 min. Afterwards, the membrane was gently dried under a stream of laboratory nitrogen by sweeping the nozzle across the sample for 2 min. The membrane was then placed in a controlled-temperature oven at 60 °C for 20 min. The resulting membranes were then removed from the frames and thoroughly rinsed. The membrane samples were referred to $M_{0.2}$, $M_{0.4}$, $M_{0.6}$, and $M_{0.8}$ for the modified and $M_{0}$ for the unmodified membrane samples.

The extent of polymerization was first evaluated in terms of the degree of grafting yield *G* using Equation (1):

(1) $$G=\left(\frac{w_{1}-w_{0}}{w_{0}}\right)\times 100$$

where *w*_0_ is the weight of the membrane before modification, and *w*_1_ is the weight of the membrane after modification.

### 2.4. Membrane Characterization

The membrane samples were characterized by scanning electron microscopy (SEM), Fourier-transformed infrared spectroscopy (FTIR), and nuclear magnetic resonance (NMR) before and after modification. The SEM was performed using an FEI Nova Nano SEM with a magnification of 1200 at a 50–100 µm distance to assess the membrane’s morphology. FTIR analysis was performed using a PerkinElmer Spectrum Two FTIR spectrometer to determine the chemical composition of the membrane surface. Solid state NMR experiments were carried out using 2.5 mm outer diameter zirconia rotors (Bruker, Karlsruhe, Germany); ^13^C NMR spectra were obtained with a Bruker AVANCE III HD 500 MHz (11.1 Tesla) standard bore spectrometer and a triple channel broadband probe (Trigamma^TM^ MAS probe), at a magic angle spinning rate of 10 kHz, frequencies of 500 MHz (^1^H) and 125.8 MHz (^13^C), and standard cross-polarization (CP) MAS techniques (^1^H π/2 pulse length 3.4 µs and ^13^C π/2 pulse length 4.0 µs, ^1^H cross polarization field 120 kHz, ^1^H–^13^C cross polarization contact time 2.0 ms, broadband SPINAL64 decoupling during signal acquisition at a ^1^H field strength of 120 kHz, recycle time 3 s, typical number of scans accumulated per spectrum ca. 10000). Chemical shifts were referenced to the downfield methylene signal from solid glycine at 43.7 ppm. The contact angles of the membranes were measured to evaluate their hydrophilicity using a OneAttension tensiometer (Biolin Scientific, Gothenburg, Sweden) with pure water at ambient temperature. Five measurement cycles were performed for each membrane, with the first cycle discarded to minimize artifacts; the average of cycles 2 to 5 and the average value ± standard deviation (*n* = 3 coupons per sample) were reported [19]. Advancing contact angles were reported as reflecting dynamic wettability by capturing the behavior of the moving contact line, thereby providing more reliable insights than static measurements [20].

### 2.5. Fouling Experiments

Solutions of the Gram-negative *E. coli* and the Gram-positive *S. aureus* bacteria were used as model microorganisms to assess the antibiofouling ability of the modified membranes. The bacterial culturing procedure was adapted from Wang et al. [21]. The highest-performing ADMH-modified membrane was then selected to evaluate microorganisms’ dynamic adhesion and to conduct organic and inorganic fouling tests in a crossflow filtration RO setup. All dynamic adhesion and fouling experiments were performed under identical hydrodynamic conditions, maintaining an applied feed pressure of 15 bar and a cross-flow velocity of 19 cm s^−1^.

### 2.5.1. Static Adhesion Test

The membrane was cut into circles 88 mm in diameter and placed under UV for 30 min. The pieces of modified and unmodified membranes were set for 3 h in contact in the incubator at 37 °C with nutrient broth (NB) containing previously prepared *E. coli* or *S. aureus* suspension, and a blank control contaminated broth solution that was not in contact with the membranes was also incubated for the same contact time for control. The membrane pieces were then removed and rinsed with saline water, and 1 mL of the suspension solution collected was diluted in series (2-10-fold). Briefly, 100 µL of diluent was plated onto nutrient agar (NA) in Petri dishes, labeled with a distinguishing pattern, and incubated at 37 °C for 20 h. The same dilution process was used for the blank control and was plated on Petri dishes. The Petri dishes were then removed, and the number of *E. coli* bacteria in contact with each membrane was determined using the plate count method. The same procedure was applied to *S. aureus* bacteria for both the unmodified and modified membrane samples.

### 2.5.2. Dynamic Adhesion Test

**Figure 2:**
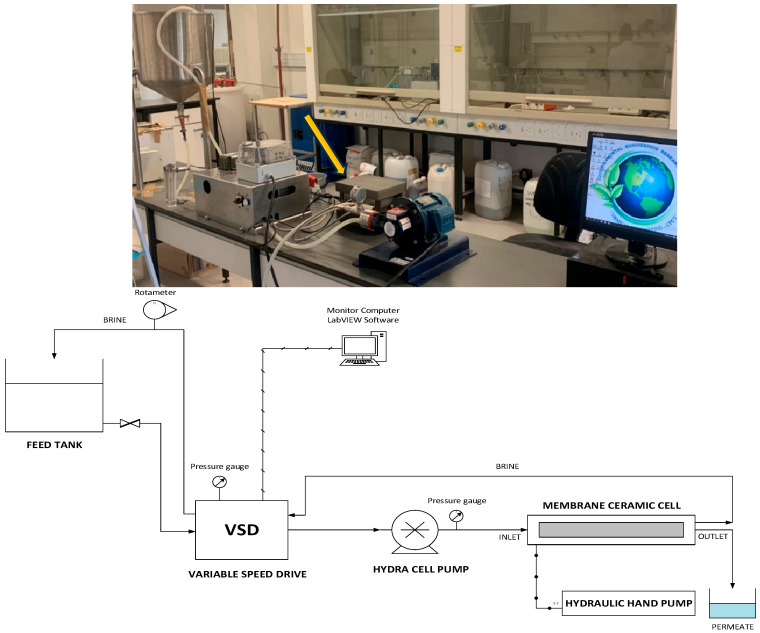
Photo (**up**) and process flow diagram (**down**) of the lab-scale flat-cell RO system.

The unmodified and ADMH-modified membranes were evaluated for their antibacterial potential using a lab-scale crossflow RO system, as illustrated in Figure 2, with bacterial suspensions as feed solution. The feed water was a synthetic solution that mimicked fouling in municipal secondary effluent, enabling an assessment of the membrane’s performance against fouling. Table 1 summarizes the fouling solutions, along with their respective concentrations, used in the dynamic adhesion tests for biofouling, as well as in the organic and inorganic fouling experimental tests. The water was pumped through the cell using a hydra-cell pump operating at 15 bar, a velocity of 19 cm s^−1^, and ambient temperature. The permeate water was discharged into a collecting beaker, and the retentate was recycled back into the feed tank. LabVIEW software (version 2022 Q3) was used to visualize every aspect of the setup and measurement data, including conductivity, flow rate, and velocity. The pressure around the cell was monitored using a hydraulic pump.

**Figure 3:**
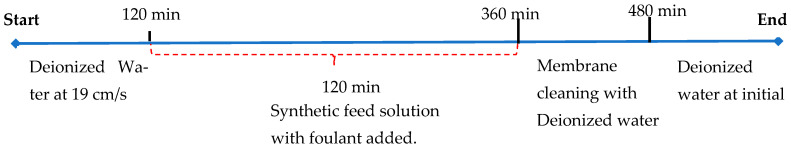
Fouling experimental timeline during membrane system performance tests.

After the cell was secured, the feed solution was added, and the pump was switched on. The system was switched on, and the LabVIEW program was started to control the operating conditions. The flux was recorded every 30 min. Figure 3 illustrates the experimental timeline followed during the membrane performance and fouling evaluation tests, comprising the initial pure water flushing stage, the fouling phase, and the final pure water cleaning step used to assess flux recovery.

### 2.6. Membrane Performance Assessment

Membrane performance was assessed by comparing the process parameters of the modified and unmodified membranes under identical operating conditions. Bacterial mortality on membranes was determined to evaluate their antibiofouling ability. The membrane water flux was assessed according to Kasongo et al. [11].


(2) $$J=\frac{Q_{P}}{A_{System}}=\frac{V}{A\times ∆t}$$

J is the flux (L/m^2^ h), $Q_{P}$ is the permeate flow, A is the surface area of the membrane surface (m^2^), *V* is the volume of the permeate (L), and ∆t is the time interval (h).

The antifouling ability of the membrane was evaluated in terms of flux decline ratio (*FDR*) and flux recovery ratio (*FRR*), as shown by Equations (3) and (4) [4]:

(3) $$FDR=\left(\frac{J_{0}-J_{t}}{J_{0}}\right)\times 100$$

(4) $$FRR=\left(\frac{J_{wf}}{J_{wi}}\right)\times 100$$

where $J_{wi}$ is the pure water flux before filtration, $J_{wf}$ the pure water flux after filtration and rinsing; $J_{0}$ the initial flux during filtration at t = 0, and $J_{t}$ the flux during filtration at a specific time t.

The salt rejection *R* was tested using NaCl solution (500 ppm) and was calculated using Equation (5) [11]:

(5) $$R=\frac{C_{f}-C_{P}}{C_{f}}\times 100$$

where $C_{f}$ is the feed conductivity (μm/cm), and $C_{P}$ the permeate conductivity (μm/cm).

The mortality ratio *R* is the number of viable bacteria in contact with the membrane for a given contact time (*A*) subtracted from the number of viable bacteria not in contact (*B*) and is given by Equation (6) [4].

(6) $$R=\left(\frac{B-A}{B}\right)\times 100.$$

All statistical analyses were performed using Design Expert. For normally distributed data, differences between groups were assessed using one-way ANOVA. The results are presented as mean, and a *p*-value of less than 0.05 was considered statistically significant.

## 3. Results and Discussion

### 3.1. ADMH Characterization

**Figure 4:**
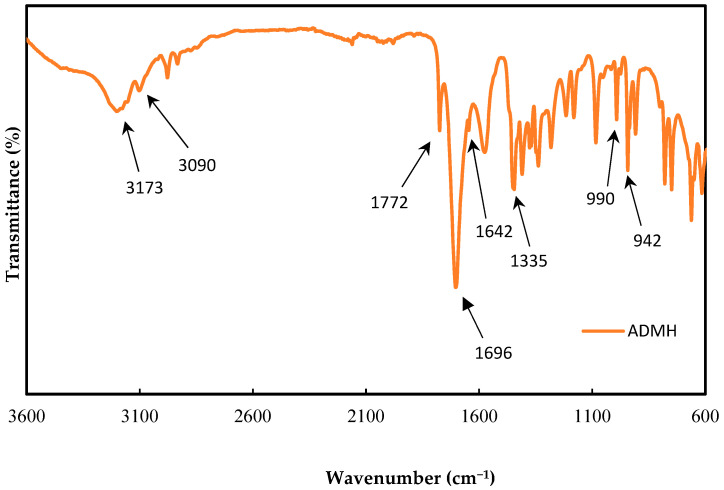
Modifying agent ADMH FTIR characteristics after synthesis.

The ADMH was characterized by FTIR using the PerkinElmer FTIR spectrum, two spectrometers with a scan range of 400–4000 cm^−1^, to confirm the chemical’s purity. Figure 4 shows the FTIR results for ADMH after synthesis.

An intense stretch at 73 cm^−1^ is due to the stretching vibration of the N−H bond and the strong pick at 1696 cm^−1^. This is due to the presence of a carbonyl group. The peak at 3090 cm^−1^ shows the strong stretching vibration of =CH bond with a weak stretching vibration peak of the C=C bond at 1642 cm^−1^. Also, the peak at 1772 cm^−1^ is due to stretching vibrations of the C=O bond peak H. Moreover, the peak at 1335 cm^−1^ is due to the in-plane bending vibration of the =CH bond, while the absorption peaks at 942 cm^−1^ and 990 cm^−1^ are present because of the out-of-plane bending of the ${=CH}_{2}$ bond [16].

### 3.2. Membrane Surface Modification and Characterization

**Figure 5:**
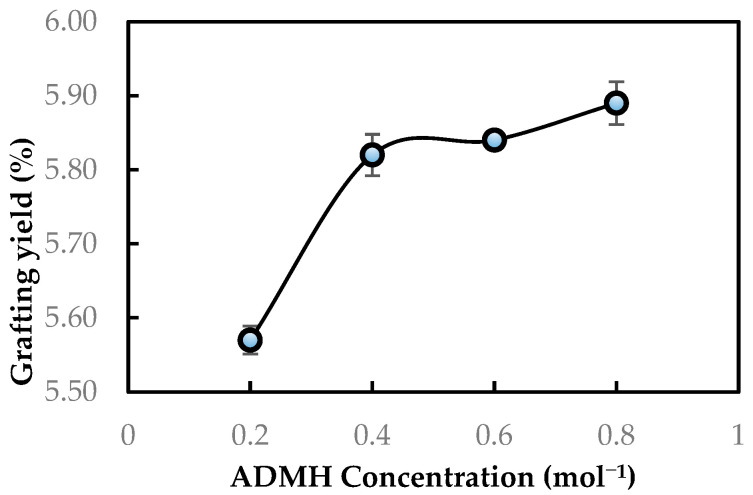
Grafting yield of ADMH on the RO membrane surface after modification.

The degree of grafting was initially evaluated using the grafting yield. Figure 5 shows the grafting yields obtained with different ADMH concentrations. It is observed that the degree of grafting increases with the grafting concentration. This is due to the greater availability of monomer molecules at the membrane surface, which affects chain propagation during graft polymerization of the RO membrane. This implies a difference in the physiochemical properties of the modified membranes, as the properties of modified membranes are a function of grafting yield, which is highly dependent on the reaction conditions, like the concentration of the modifying agent [11].

**Figure 6:**
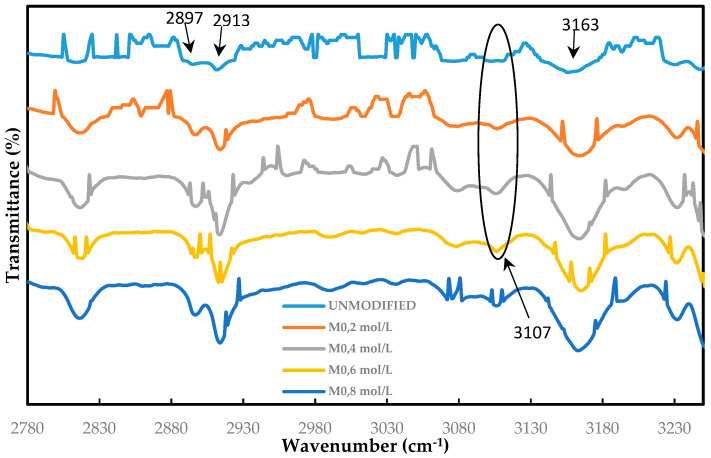
FTIR graphs for the unmodified, M_0_, M_0.2mol/L_, M_0.4mol/L_, M_0.6mol/L_, and M_0.8mol/L_ (modified membranes with 0.2 mol L^−1^, 0.4 mol L^−1^, 0.6 mol. L^−1^, and 0.8 mol L^−1^, respectively) in the wavenumber range of 2780–3230 cm^−1^.

The effect of surface-grafted ADMH on the membrane surface was investigated using FTIR, SEM, and NMR spectroscopy. Figure 6 shows the FTIR spectrum of the unmodified and ADMH-grafted membranes. The appearance of the characteristic peaks is similar to the ones reported by Wei et al. [16]. The FTIR spectra of ADMH-grafted membranes clearly show the formation of a new band in the 3107 cm^−1^, which is a C=O bond in ADMH. The band becomes more visible as the ADMH concentration increases. Indeed, this observation suggests that ADMH has been successfully grafted onto the aromatic polyamide RO membrane surface, and the degree of grafting is more significant with increasing grafting concentration [12]. The peaks at 2897 cm^−1^ and 2913 cm^−1^ are like the C−O (amide I) stretching, the hydrogen-bonded C−O (amide I) stretching, and N−H (amide II) in-plane bending, respectively. These stretchings are characteristics of polyamide in the barrier layer. The intense stretching at 3163 cm^−1^ is an Ar−O−Ar stretching; Ar is used to denote an aromatic ring, which is also characteristic of the membrane support layer [22]. These findings are consistent with the findings reported by Wei et al. [16] and, therefore, confirm the successful graft of ADMH on the membrane surface [12].

**Figure 7:**
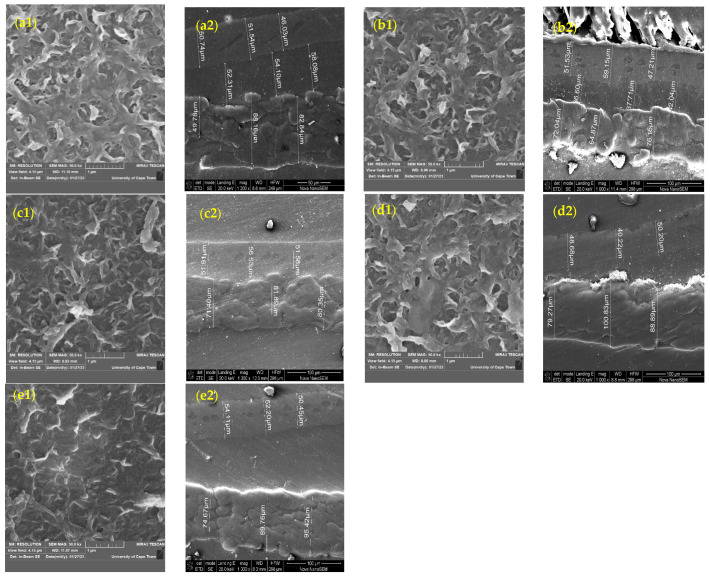
SEM images of the unmodified and ADMH-grafted membranes (top view and cross-section): (**a1**,**a2**) unmodified membrane, (**b1**,**b2**) 0.2 mol L^−1^ ADMH, (**c1**,**c2**) 0.4 mol L^−1^ ADMH, (**d1**,**d2**) 0.6 mol L^−1^ ADMH, (**e1**,**e2**) 0.8 mol L^−1^ ADMH.

SEM analysis was performed to assess the membrane morphology before and after modification. Figure 7 shows the top-view SEM images of the RO membrane surface at 1 µm magnification and the cross-sectional images of the membranes on the left and the right, respectively. The typical multi-layered ridge-and-valley structure was observed in the unmodified membranes in Figure 7(a1). Indeed, the top-view SEM images of the modified membranes show partial or complete filling of the XLE-4040 membrane pores by grafting chains. For the modified membranes, a less uniform appearance was observed, with irregularities in the form of matter. This observation is evident for the 0.6 mol/L grafted membrane shown in Figure 7(d1). The irregularities observed on the membrane surface confirm ADMH’s adhesion, as suggested by Vatanpour et al. [23].

Also, the irregularities are more prominent with increasing concentration. The membrane surface modification caused the valleys of the commercial membranes to be filled with the grafting polymer ADMH.

**Figure 8:**
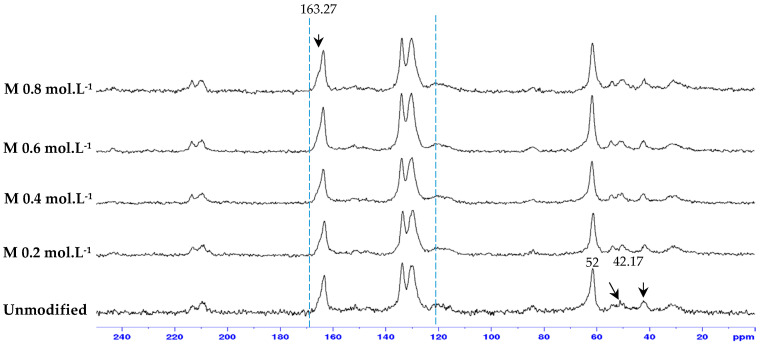
Solid C^13^ CPMAS NMR results for unmodified and all ADMH-modified membranes.

Figure 7 also presents a cross-sectional view of the unmodified and grafted membrane samples. The cross-sectional thickness of the unmodified membrane is 88.16 µm, which differs from that of the modified membranes. The ADMH-grafted membranes exhibit new characteristics in the top layer due to the addition of a thin layer. This confirms a successful modification of the membranes by ADMH grafting. Nuclear magnetic resonance spectroscopy was also used to analyze the structural alterations in the virgin reverse osmosis membrane and the modified membranes, as shown in Figure 8.

The carbonyl peak at 165 ppm belongs to the reverse osmosis membrane. Moreover, the chemical shift at 5.6–5.8 ppm was due to the methine proton in the pendant allyl groups. The spectrum of the neat bulk membrane displays resonances at 110 to 165 ppm, which belong to the reverse osmosis membrane. Liu et al. [22] presented a nuclear magnetic resonance of ADMH and identified the elemental bonds of the agent. The pick-in at 52 ppm represents the (C (CH_3_)_2_) of the ADMH. The peak at 42.17 ppm corresponds to the (=CHCH_2_) bond in ADMH, which increases with increasing concentration. The pick at 130.4 ppm is (=CHCH_2_), while the pick at 163.27 ppm is the (C=O) bond in the ADMH structure. The variations observed from the membrane sample pick confirm successful graft polymerization [22].

**Figure 9:**
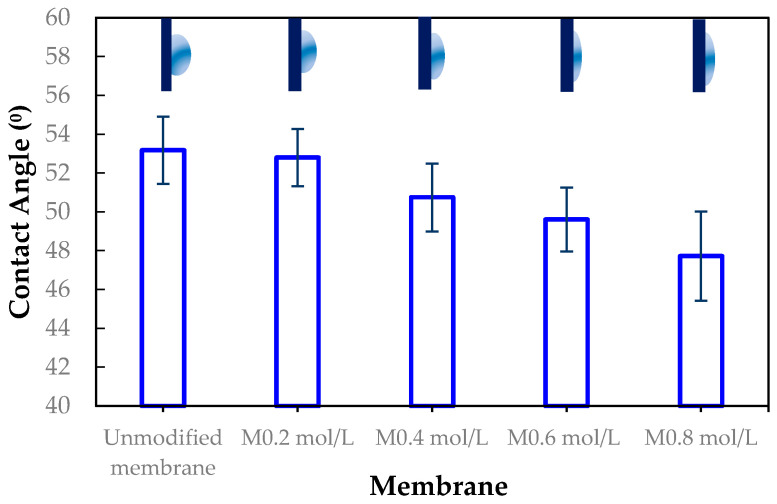
Contact angle measurements of unmodified and ADMH-modified RO membranes at different grafting concentrations (0.2–0.8 mol L^−1^).

The membrane surfaces were characterized using advancing contact angle measurements to assess hydrophilicity. As shown in Figure 9, the contact angle decreased progressively with increasing ADMH concentration, from 53.17° ± 1.73° for the unmodified membrane to 47.72° ± 2.29° for the 0.8 mol/L-modified membrane, with intermediate values of 52.80° ± 1.48°, 50.74° ± 1.75°, and 49.61° ± 1.65° for 0.2, 0.4, and 0.6 mol/L modifications, respectively. This trend indicates that ADMH grafting effectively enhances surface hydrophilicity. The introduction of hydrophilic amine and hydroxyl groups increases surface polarity, promoting water affinity and thereby lowering the contact angle [5]. These findings align with previous reports indicating that the introduction of hydrophilic materials to the membrane surface reduces contact angles, thus improving membrane wettability [16].

### 3.3. Biofouling Resistance

**Figure 10:**
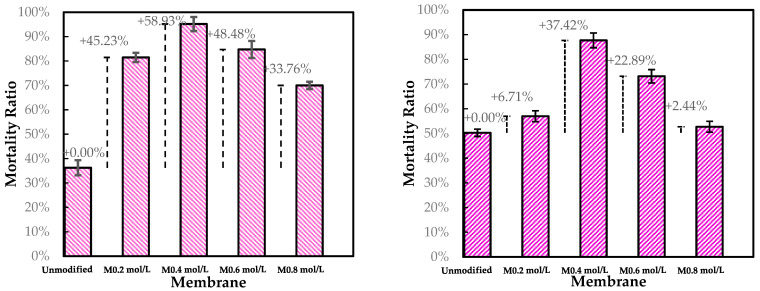
Static adhesion test results for unmodified and modified membranes against *E. Coli* (**right**) and *S. Aureus* (**left**).

Figure 10 presents the mortality ratio of modified membranes compared to pristine membranes tested with an *E. coli* solution. The membranes M0.2 mol L^−1^ and M0.4 mol L^−1^ (modified with 0.2 mol L^−1^ and 0.4 mol L^−1^ concentrations of ADMH, respectively) exhibits improved mortality rates of 45.23% and 58.93%, respectively. The membranes M 0.6 mol L^−1^ and M 0.8 mol L^−1^ (modified with 0.6 mol L^−1^ and 0.8 mol L^−1^ concentrations of ADMH, respectively) exhibit mortality ratios of 48.48% and 33.76%, respectively. One-way ANOVA showed a significant difference between unmodified and modified membranes when considering all mortality ratios (*p* = 0.0253). The grafting of ADMH onto the membrane introduces membrane functional groups that enable interfacial contact-killing, thereby reducing biofouling. Bromberg et al. [24] demonstrated that N-halamine/hydantoin coatings provide durable contact-killing. Xiao et al. [25] showed that grafting molecules, such as 2-aminoethanethiol, tune interactions with foulants while maintaining permeance. This aligns with the present findings, where moderate ADMH concentrations enhanced hydrophilicity and foulant repellence without significantly compromising membrane permeability.

It was observed that the membrane M0.4 mol L^−1^ had the highest improvement of 58.93% and appeared to be the optimal modified membrane against *E. coli*.

Compared to *E. coli*, the mortality rate of the modified membranes is lower against *S. aureus*. Due to electrostatic interactions, Gram-negative bacteria such as *E. coli* are repelled by the negatively charged surface of modified membranes, contrary to *S. aureus*, which has a positively charged outer surface [26]. This may also be explained by the fact that Gram-positive bacteria can form more robust biofilms, which might offer better protection against modified membrane surfaces and hence decrease mortality.

**Figure 11:**
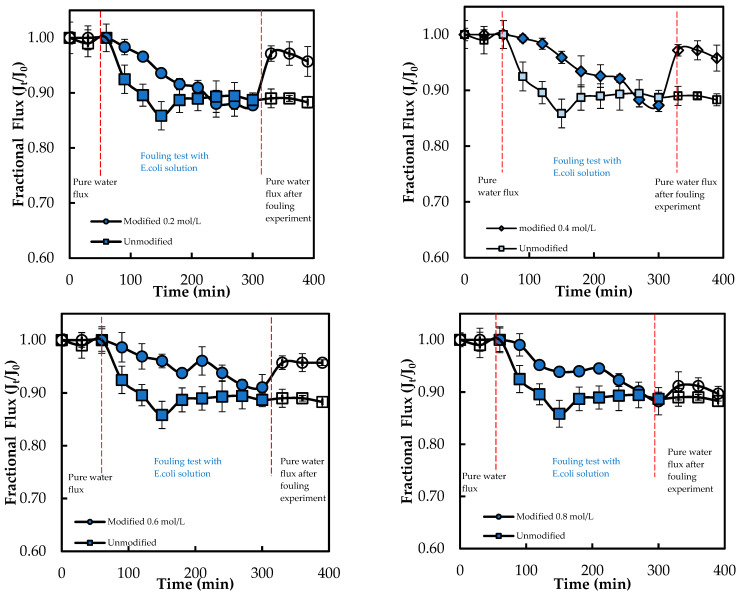
Flux of ADMH-grafted membranes compared to the unmodified membrane during dynamic fouling tests with *E. coli* solution using an RO system.

Moreover, the membrane M0.4 mol L^−1^ shows the highest improvement of 37.42% and appears to be the optimal modified membrane against *E. coli*. The success of the graft polymerization of ADMH is observed by the behavior of the 0.4 mol/L membranes. However, the membrane sample at 0.8 mol L^−1^ exhibited the lowest improvement, which could be due to the alteration of the membrane, resulting in a reduction of electrostatic interactions necessary for an effective biofouling-resistant surface [12]. The improved biofouling resistance activity observed at moderate ADMH concentrations (0.2–0.4 mol L^−1^) can be attributed to adequate surface coverage that maintains the accessibility of active groups while preserving permeability and electrostatic repulsion. At higher concentrations (>0.6 mol L^−1^), however, excessive polymer deposition may cover functional groups, increase hydrogen bonding interactions, and hinder water transport [27]. Furthermore, it is observed that although surface hydrophilicity increases with ADMH concentration, antibiofouling performance peaks at 0.4 mol·L^−1^ and declines at 0.8 mol·L^−1^. This suggests that enhanced hydrophilicity alone is not decisive; at moderate concentrations, the denser hydrophilic groups promote a stable hydration layer that resists adhesion, whereas excessive concentrations cause surface heterogeneity, amplifying hydrophilicity but creating sites that facilitate microbial attachment and stronger foulant interactions [28]. The flux trend decreases over time, as shown in Figure 11, confirming the presence of microbial fouling, which is responsible for the decline in flux. The modified membranes exhibited higher fluxes than the unmodified ones, likely due to improvements in surface properties resulting from ADMH grafting. The anti-biofouling properties of ADMH are attributed to the strong bond formed on the membrane surface, which affects the flux of the membranes [16].

**Figure 12:**
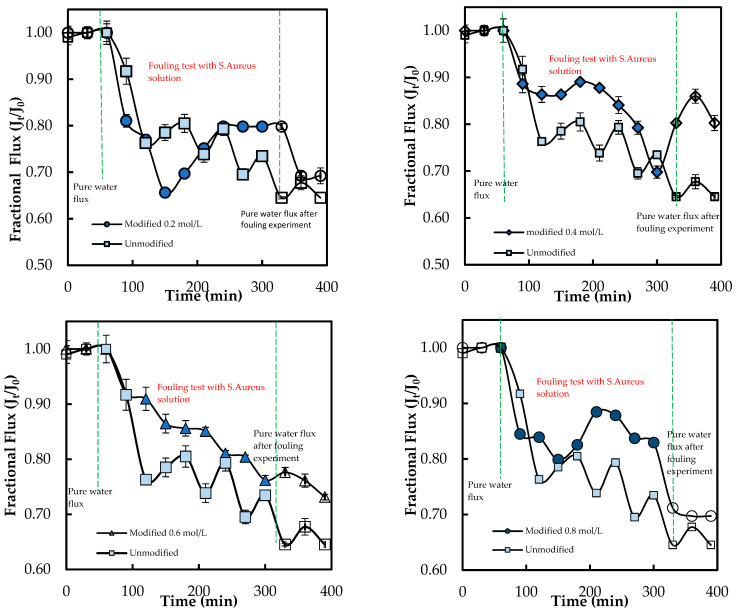
Flux of ADMH-grafted membranes compared to the unmodified membrane during dynamic fouling tests with *S. aureus* solution using an RO system.

Figure 12 shows that the flux decreases over time, confirming the presence of microbial fouling by *S. aureus*. The overall flux trend of the modified membranes is similar to that of the unmodified membrane. However, the fluxes of the modified membranes are slightly lower than those of the unmodified membrane, with a value of 33.58%, while the highest flux is 54.47% for the unmodified membrane. This observation could be due to the polymerization of the aromatic layer of the grafted membrane becoming more compact due to heat treatment, which is responsible for increased water resistance [17].

The performance results of the modified and unmodified membranes are presented in Table 2. The salt rejection for the modified membranes was 80.99%, 81.62%, 80.81%, and 80.33% for the M_0.2mol/L_, M_0.4mol/L_, M_0.6mol/L_, and M_0.8mol/L_ membranes, respectively. It was observed that the salt rejection increased after surface modification.

Moreover, the membrane permeability of the modified membranes with ADMH concentrations of 0.2 mol L^−1^, 0.4 mol L^−1^, 0.6 mol L^−1^, and 0.8 mol L^−1^ was 9.52 L m^2^ h^−1^.bar, 9.51 L m^2^ h^−1^. bar, 9.18 L m^−2^ h^−1^.bar, and 9.02 L m^−2^ h^−1^.bar. A decrease in permeability was observed with increased ADMH concentration, attributed to the addition of a layer on the membrane surface after modification. Wei et al. [16] reported that grafting time affects permeability by altering the membrane’s hydrophilicity. Moreover, according to Wang et al. [21], modifying the agent molecules at the membrane surface decreases hydrogen bonding between the carboxylic and amine groups of the polyamide layer, resulting in a higher flux for the modified membrane.

After 240 min of fouling, the lowest *FDR* value of 8.91% with *E. coli* was observed for M_0.6mol/L_, and the highest *FRR* value of 96.88% was observed for M_0.2mol/L_. Furthermore, the M_0.2mol/L_ membrane had the lowest *FDR* value of 3.72%, while the M_0.6mol/L_ membrane had the highest FRR value of 70.15% when tested with *S. aureus*. The decline in fluxes of ADMH-grafted membranes may have been caused by the recovery of the inter-chain hydrogen [17].

### 3.4. Organic and Inorganic Fouling Resistance

#### 3.4.1. Organic Fouling Resistance

**Figure 13:**
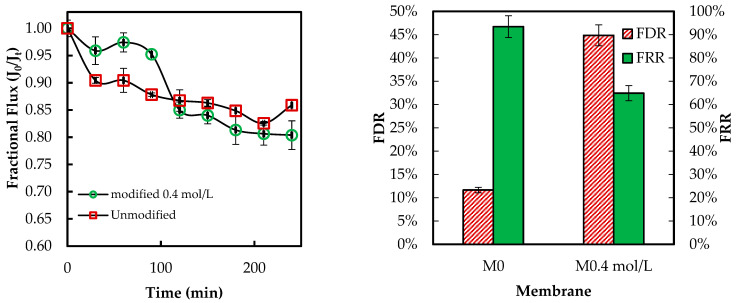
Membranes’ antifouling properties during fouling tests with organic foulant solutions.

Figure 13 below presents the flux behavior of the ADMH-grafted membranes during the organic fouling test using humic acid solution.

The overall fluxes through the modified membrane follow a pattern similar to those through the unmodified membrane. The *FDR* values are 11.66% and 44.87% for the unmodified and modified membranes. The *FRR* values are 93.47% and 64.89% for the unmodified and modified membranes, respectively. The modified membrane exhibited a lower flux than the unmodified membrane. This may be explained by the fact that ADMH’s functional groups can form hydrogen bonds with the organic foulant, thereby increasing their adhesion to the membrane surface. In contrast to the adhesion of microorganisms, the grafting of ADMH may have provided more sites for organic molecules to adhere to, thereby promoting fouling. Similar trade-offs have been reported, where improvements in one property may compromise others [27]. Da’na et al. [29] further confirmed that the polarity and type of surface functional groups are key factors governing fouling interactions.

#### 3.4.2. Inorganic Fouling Resistance

**Figure 14:**
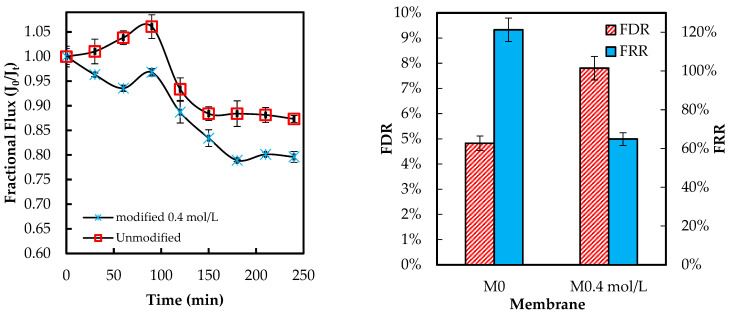
Membranes’ antifouling properties during fouling tests with inorganic foulant solutions.

Figure 14 below presents the flux behavior of the ADMH-grafted membranes during the inorganic fouling test using sodium bicarbonate (NaHCO_3_).

The *FDR* values are 4.82% and 11.34%. 87% for the unmodified and modified membranes, respectively. Similarly, the unmodified membrane had a higher FRR than the modified membrane. The slight decline in resistance to both organic and inorganic fouling indicates that improved hydrophilicity was insufficient to prevent fouling, with foulant surface affinity exerting a more significant influence on the fouling mechanism. The decline in resistance to organic and inorganic fouling can be attributed to increased surface polarity from the amide and hydantoin groups, which facilitate hydrogen bonding and dipole interactions with organic molecules and bicarbonate species. Hence, functional groups and inorganic ions can jointly influence organic and inorganic fouling mechanisms [25], while the same reactive surface enhances the contact-killing of microorganisms.

Mallah et al. [30] reported that introducing hydrophilic nanocomposites enhanced permeability and reduced microbial attachment, but slightly increased organic adsorption due to new polar interaction sites. Similarly, Bromberg et al. [24] found that non-leaching N-halamine coatings maintained vigorous antibacterial activity yet exhibited moderate protein adsorption, while Xiao et al. [25] also demonstrated that grafted functional groups can change foulant interaction energies, influencing adsorption dynamics on RO membranes.

Despite a slight change in *FDR*, particularly with the modified membranes, the F-test (*p* = 0.27) indicates that this change is not statistically significant.

## 4. Conclusions

This study assessed the fouling resistance of reverse osmosis membranes after graft polymerization of ADMH. ADMH was used at different concentrations to enhance the membrane surface antibiofouling properties and was further tested for its resistance to organic and inorganic fouling.

- SEM, FTIR, NMR, and contact angle confirmed ADMH grafting. SEM revealed surface irregularities and a distinct top-layer formation, FTIR showed new peaks, including a C=O bond at 3107 cm^−1^, and NMR detected characteristic ADMH signals with a ppm shift at 130.4 (═CHCH_2_). The contact angle analysis showed that grafting reduced surface hydrophobicity, particularly at moderate ADMH concentrations, supporting enhanced water membrane interactions.
- Biofouling tests showed improved mortality ratios against both *E. coli* (45.23%, 33.76%, 48.48%, and 58.93% for M_0.2mol/L_–M_0.8mol/L_) and *S. aureus* (6.71%, 37.42%, 22.89%, and 2.44% for M_0.2mol/L_–M_0.8mol/L_).
- The biofouling resistance test demonstrated improved biofouling resistance properties after the grafting of ADMH on the membrane surface. The M_0.2mol/L_ and M_0.6mol/L_ membranes exhibited the lowest FDR values of 3.72% against *S. aureus* and 8.91% against *E. coli*, respectively, with FRR values of 69.23% and 96.88%. The M_0.4mol/L_ showed an FRR value of 94.27% against *E. coli* bacteria and 59.70% against *S. aureus*.
- The study reveals the impact of ADMH on improving membranes’ biofouling resistance properties. However, after ADMH grafting, the membrane showed a slight decrease in resistance to both organic and inorganic fouling.
- Despite increasing hydrophilicity with ADMH concentration, fouling resistance peaked at 0.4 mol L^−1^ and slightly declined at 0.8 mol L^−1^, indicating that surface heterogeneity can override hydrophilicity in governing foulant adhesion.

### Challenges and Recommendations

This study demonstrated the effectiveness of ADMH grafting in enhancing membrane biofouling resistance and limitations to organic and inorganic fouling; however, future studies could assess long-term stability and process scalability under different operating conditions. Extending the approach through hybrid surface modifications or nanoparticle incorporation may further optimize resistance to organic, inorganic, and microbial fouling while preserving high permeability and salt rejection.

## Figures and Tables

**Table 1:** Feed and fouling solutions used in this study.

Test Type	Model Solution/Foulant	Concentration
Permeability	Deionized water	≈5 μS m^−1^
Salt rejection	Sodium chloride	500 ppm
Organic fouling	Humic acid	100 mg L^−1^
Inorganic fouling	Sodium bicarbonate	100 mg L^−1^
Biofouling	Escherichia coli	≈1.5 × 10^8^ CFU mL^−1^
Biofouling	Staphylococcus aureus	≈8.1 × 10^8^ CFU mL^−1^

**Table 2:** Mortality ratio, FDR, and FRR values of ADMH grafted membranes.

	Salt Rejection (%)	Permeability (L/m^2^ h Bar)	FDR (%)		FRR (%)	
			*E. coli*	*S. aureus*	*E. coli*	*S. aureus*
M_0_	76 ± 1.27%	7.26 ± 0.15	11.29 ± 0.72%	26.53 ± 1.12%	82.26 ± 0.89	48.39 ± 1.41%
M_0.2mol/L_	81 ± 0.58%	9.52 ± 0.98	12.24 ± 0.53%	3.72 ± 2.03%	96.88 ± 1.38	69.23 ± 1.66%
M_0.4mol/L_	82 ± 1.01%	9.51 ± 0.56	12.68 ± 0.81%	30.21 ± 0.93%	94.27 ± 1.77	59.70 ± 1.99%
M_0.6mol/L_	81 ± 0.91%	9.18 ± 0.44	8.91 ± 0.89%	23.79 ± 1.51%	96.88 ± 1.26	70.15 ± 1.82%
M_0.8mol/L_	80% ± 0.43	9.51 ± 0.67	11.74 ± 0.13%	17.03 ± 1.69%	88.40 ± 2.46	65.67 + 1.91%

## Data Availability

The data supporting the findings of this study are available from the corresponding author upon reasonable request.
